# Trends in surgical and catheter interventions for isolated congenital shunt lesions in the UK and Ireland

**DOI:** 10.1136/heartjnl-2018-314428

**Published:** 2019-02-16

**Authors:** Mehreen Farooqi, John Stickley, Rami Dhillon, David J Barron, Oliver Stumper, Timothy J Jones, Paul F Clift, William J Brawn, Nigel E Drury

**Affiliations:** 1 Paediatric Cardiac Surgery, Birmingham Children’s Hospital, Birmingham, UK; 2 Institute of Cardiovascular Sciences, University of Birmingham, Birmingham, UK; 3 Paediatric Cardiology, Birmingham Children’s Hospital, Birmingham, UK; 4 Adult Congenital Cardiology, Queen Elizabeth Hospital Birmingham, Birmingham, UK

**Keywords:** congenital heart disease surgery, interventional cardiology and endovascular procedures, atrial septal defect, patent ductus arteriosus, ventricular septal defect

## Abstract

**Objective:**

To evaluate time trends in the use of catheter and surgical procedures, and associated survival in isolated congenital shunt lesions.

**Methods:**

Nationwide, retrospective observational study of the UK National Congenital Heart Disease Audit database from 2000 to 2016. Patients undergoing surgical or catheter procedures for atrial septal defect (including sinus venosus defect), patent foramen ovale, ventricular septal defect and patent arterial duct were included. Temporal changes in the frequency of procedures, and survival at 30 days and 1 year were determined.

**Results:**

40 911 procedures were performed, 16 604 surgical operations and 24 307 catheter-based interventions. Transcatheter procedures increased over time, overtaking surgical repair in 2003–2004, while the number of operations remained stable. Trends in interventions differed according to defect type and patient age. Catheter closure of atrial septal defects is now more common in children and adults, although surgical interventions have also increased. Patent foramen ovale closure in adults peaked in 2009–2010 before falling significantly since. Surgery remains the mainstay for ventricular septal defect in infants and children. Duct ligation is most common in neonates and infants, while transcatheter intervention is predominant in older children. Excluding duct ligation, survival following surgery was 99.4% and ≈98.7%, and following catheter interventions was 99.7% and ≈99.2%, at 30 days and 1 year, respectively.

**Conclusions:**

Trends in catheter and surgical techniques for isolated congenital shunt lesions plot the evolution of the specialty over the last 16 years, reflecting changes in clinical guidelines, technology, expertise and reimbursement, with distinct patterns according to lesion and patient age.

## Introduction

Surgical repair has been the mainstay of treatment for structural congenital heart defects in children and adults for over half a century. However, over the last 20 years, interventional catheter procedures have emerged as an alternative to surgery for some lesions, with the potential for anatomical correction but reduced early morbidity.^1^ With advances in technology, increased availability and refinement of indications, catheter interventions have become the procedure of choice in many centres for the treatment of isolated shunt lesions: atrial septal defect (ASD), patent foramen ovale (PFO), ventricular septal defect (VSD) and patent arterial duct (PDA). The relative use of surgical or catheter procedures for different lesions, age groups and centres is unknown.

The National Congenital Heart Disease Audit (NCHDA) is a national database that collects validated electronic data on all cardiac surgical and therapeutic cardiac catheterisation procedures from specialist centres in the UK and Ireland.^2^ Their website contains a public record of the number of procedures reported since 2000, by year, procedure, age group and centre. We therefore used NCHDA data to analyse trends in surgical and catheter procedures for the management of isolated shunt lesions.

## Methods

Surgical and catheter procedures for the treatment of isolated shunt lesions from 1 April 2000 to 31 March 2016 were identified from the NCHDA database.^2^ As an audit of publicly available anonymised summary data, National Health Service (NHS) ethical approval was not required. Data were collected by procedural code, financial year, age group and centre in March 2018. Catheter codes for ‘ASD closure (catheter)’, ‘PFO closure (catheter)’, ‘VSD closure (catheter)’ and ‘PDA closure (catheter)’ were used. Surgical codes for ‘ASD repair’, ‘Sinus venosus ASD and/or PAPVC repair’, ‘VSD repair’, ‘Multiple VSD closure’ and ‘PDA ligation (surgical)’ were collected; codes for more complex procedures such as ‘Atrioventricular septal defect (partial) repair’ were not included. Patient age was defined according to standard reported categories: neonate (0–30 days), infant (31 days–1 year), child (1–16 years) and adult (>16 years). Survival at 30 days and 1 year were recorded; these are calculated from the dates of intervention and death, with mortality tracking from the Office for National Statistics (ONS). Missing cases were combined with known survivors to calculate ‘assumed alive’ as an upper limit of survival probability at 1 year. The number of live births and total UK population each year over the same period were obtained from ONS.^3 4^

### Statistical methods

Statistical analysis was performed using Excel (Microsoft, Redmond, Washington, USA) and R (https://www.r-project.org/). Categorical data were expressed as counts and percentages. Fisher’s exact test was used to compare categorical variables and trends in interventions were assessed using Kendall’s method.

## Results

A total of 40 911 procedures for isolated shunt lesions in the UK and Ireland were recorded between 2000 and 2016, 16 604 surgical operations and 24 307 catheter-based interventions. Paediatric procedures were performed at 16 centres, adult surgery at 33 centres and adult catheter interventions at 34 centres, including procedures in patients aged >16 years performed at ‘paediatric’ centres. The number of sites reporting data to NCHDA by year is shown in the online supplementary eFigure A.

10.1136/heartjnl-2018-314428.supp1 Supplementary file 1

**Figure 1:**
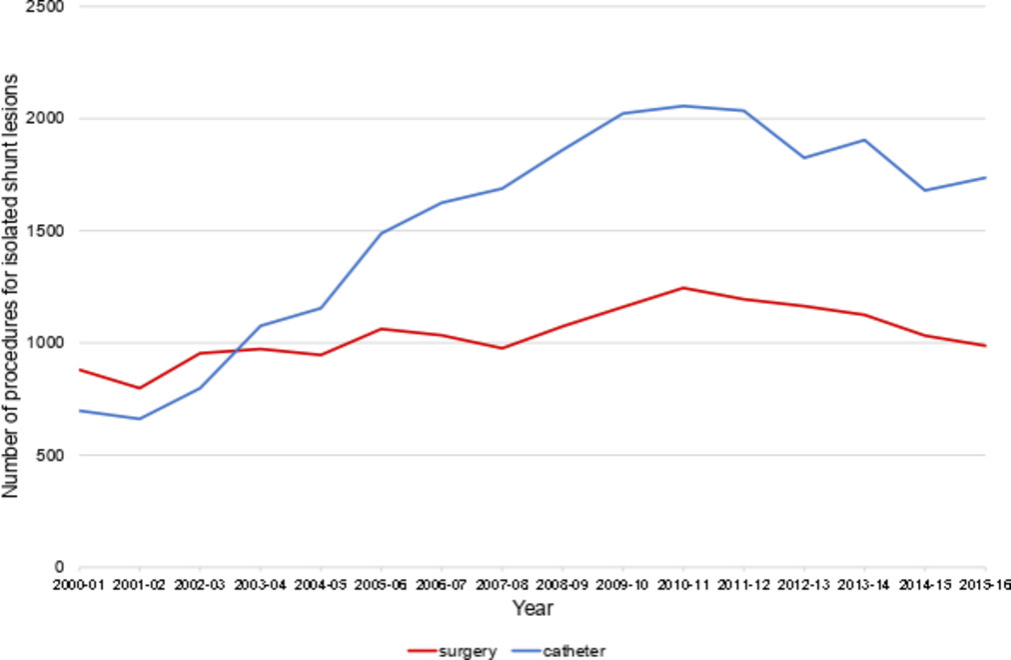
Trends in the total number of surgical and catheter procedures performed for isolated shunt lesions.

**Figure 3:**
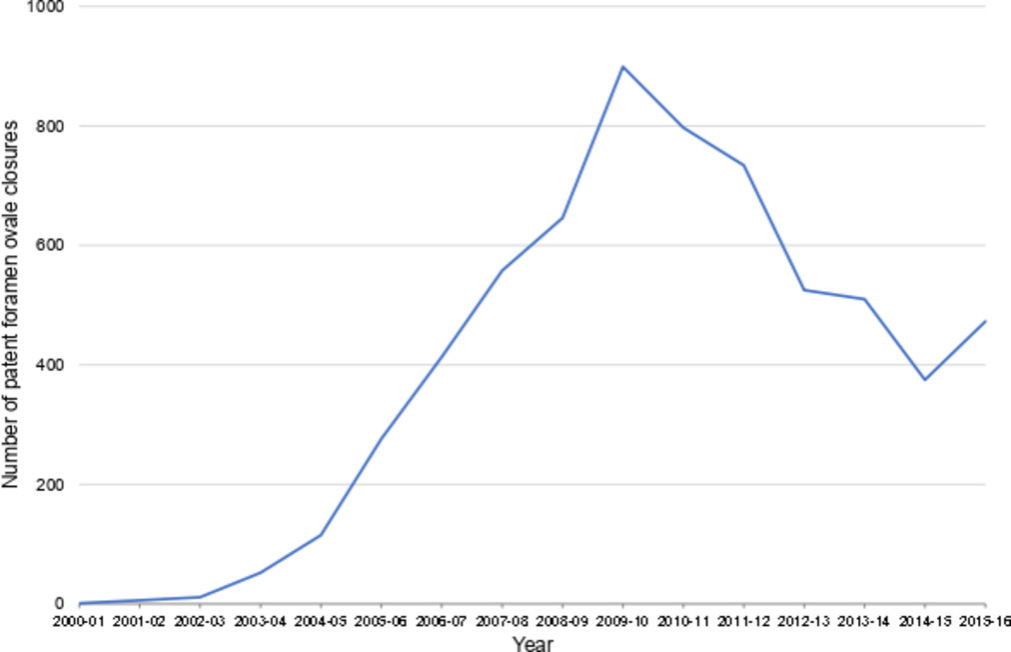
Trends in closure of patent foramen ovale in adults by catheter device.

The number of catheter procedures per year has increased (τ=0.63, p<0.001), overtaking surgical repair in 2003–2004 and peaking at 2056 interventions in 2010–2011 (figure 1); the subsequent fall is consistent with the reduction in adult PFO device closures (figure 3). Surgical procedures also rose during the study period (τ=0.5, p<0.001), but have plateaued at around 1000 operations per year. The rate of live births increased by an average of 0.9% per year and total UK population grew by 0.7% per year over the same period. Summary tables for each defect by intervention, age group and year are available in the online supplementary eTable 1–7 .

Overall survival following surgical repair was 99.1% at 30 days and up to 97.8% at 1 year; after excluding PDA ligation, which accounted for 80% of postoperative deaths, survival for ASD/VSD repair was 99.4% and up to 98.7% at 30 days and 1 year, respectively. Following catheter interventions, overall survival was 99.7% at 30 days and up to 99.2% at 1 year, although outcome was significantly worse after VSD device closure than surgical repair (95.7% vs 99.4% at 30 days, p<0.001) and following PDA ligation than device occlusion (95.6% vs 99.8%, p<0.001). A breakdown for each lesion is shown in table 1 and by year quartile is shown in the online supplementary  etable 8 .

**Table 1:** Number of cases and survival at 30 days and 1 year following intervention, by defect and intervention

Defect	Intervention	Cases	30-day survival			1-year survival			
			Alive	Dead	Unknown	Known alive	Dead	Unknown	Assumed alive*
ASD, no. (%)	Catheter	9263	9245 (99.8)	14 (0.2)	4 (0.04)	9263 (86.3)	64 (0.7)	1205 (13.0)	9199 (99.3)
	Surgery	5755	5727 (99.5)	26 (0.45)	2 (0.03)	4733 (82.2)	49 (0.9)	973 (16.9)	5706 (99.1)
PFO, no. (%)	Catheter	6465	6451 (99.8)	14 (0.2)	0	6118 (94.6)	39 (0.6)	308 (4.8)	6426 (99.4)
VSD, no. (%)	Catheter	604	578 (95.7)	25 (4.1)	1 (0.17)	489 (81.0)	33 (5.5)	82 (13.6)	571 (94.5)
	Surgery	5623	5588 (99.4)	33 (0.6)	2 (0.04)	4390 (78.1)	102 (1.8)	1131 (20.1)	5521 (98.2)
PDA, no. (%)	Catheter	7975	7962 (99.8)	13 (0.2)	0	6533 (81.9)	48 (0.6)	1394 (17.5)	7927 (99.4)
	Surgery	5226	4997 (95.6)	228 (4.4)	1 (0.02)	3874 (74.1)	576 (11.0)	776 (14.8)	4650 (89.0)
Overall, no. (%)	Catheter or surgery	40 911	40 548 (99.1)	353 (0.9)	10 (0.02)	34 131 (83.4)	911 (2.2)	5869 (14.3)	40 000 (97.8)

*Assumed alive=known alive+unknown.

ASD, atrial septal defect; PDA, patent arterial duct; PFO, patent foramen ovale; VSD, ventricular septal defect.

### Atrial septal defect

**Figure 2:**
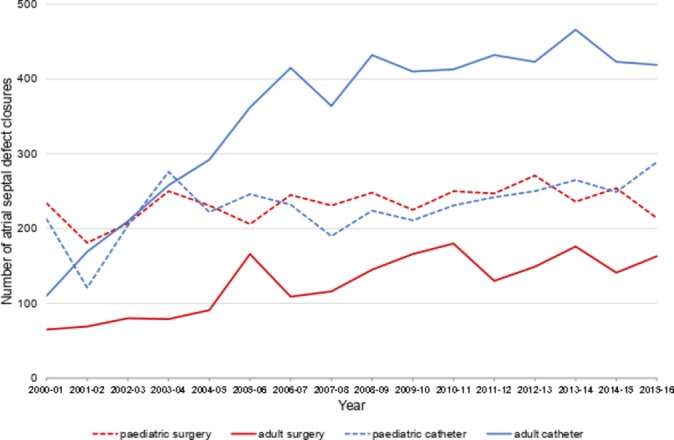
Trends in closure of atrial septal defects by surgical repair or catheter device.

ASD device closures more than doubled from 323 in 2000–2001 to 708 in 2015–2016, primarily due to a rise in procedures performed in adults during the first half of the series, increasing from 110 in 2000–2001 to 432 in 2008–2009 (figure 2). There was not a corresponding fall in surgical ASD closures, which remained between 370 and 430 per year in recent years. While most surgical ASD closures are performed during childhood, catheter interventions have increased in this cohort, such that device closure is now the more common intervention. The rise in adult catheter device closures over the first 6 years occurred at established congenital heart disease centres (τ=1, p=0.008) with no significant increase over the last decade (τ=0.36, p=0.18), as shown in the online supplementary eFigure B.

Comparing the ratio of catheter with surgical ASD interventions, there was a rise in the proportion of device closures from 2000–2001 to 2006–2007 (τ=0.52, p=0.006) reaching a plateau over the last decade (see online supplementary eFigure C). Of note, the number of reported surgical closures of sinus venosus defects increased significantly from 29 (5.4% of ASDs, 11.6% of ASD surgery) in 2001–2002 to 129 (11.3% of ASDs, 31.3% of ASD surgery) in 2013–2014 (τ=0.8, p<0.001) (see online supplementary eFigure D).

### Patent foramen ovale

PFO device closures are almost exclusively performed in adults (98.9%). There was a marked rise in the number of procedures performed, from 1 case in 2000–2001 to a peak of 899 cases in 2009–2010, followed by a fall to around 450 per year in recent years (figure 3).

### Ventricular septal defect

**Figure 4:**
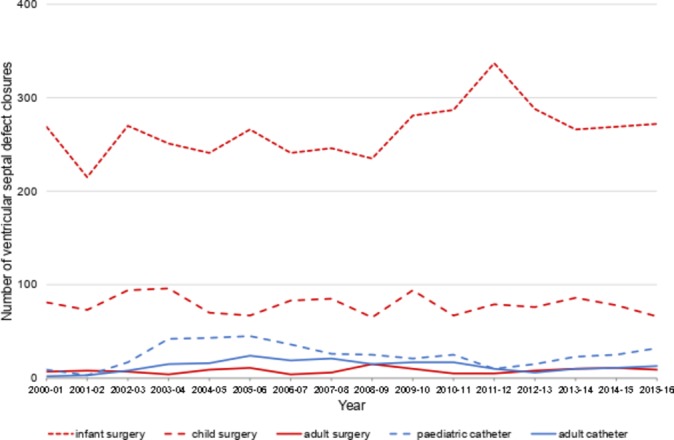
Trends in closure of ventricular septal defects by surgical repair or catheter device.

Surgical VSD repair is performed more commonly than catheter device closure, with the latter accounting for only 9.7% of VSD interventions. Most VSDs are closed surgically during infancy with around a quarter closed after 1 year of age but very few in adulthood (figure 4). Catheter procedures are most commonly performed in children with a peak at 42 cases/year in 2004–2005, with most of these procedures performed at three centres (Birmingham, Bristol, Royal Brompton). Adults are the only group in whom device closure is more common than surgery but the absolute numbers of interventions are low.

### Patent arterial duct

**Figure 5:**
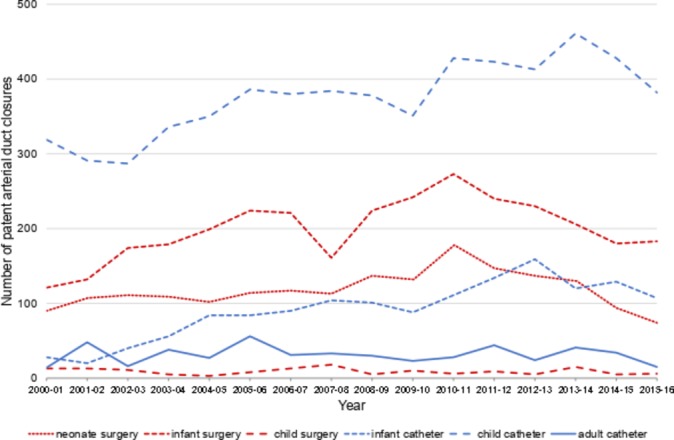
Trends in closure of patent arterial ducts by surgical ligation or catheter occlusion.

Most PDA interventions are performed by catheter in children over 1 year of age (figure 5) in whom isolated surgical ligation is rare. The number of device occlusions in infants has also increased (τ=0.62, p=0.001). Surgical PDA ligations in neonates have reduced by 58.2% in the last 5 years, from a peak of 177 cases in 2010–2011 to 74 cases in 2015–2016, with a similar fall (273–183, 33.0%) in infants. PDA intervention is uncommon in adults.

## Discussion

In this study of the UK national database for congenital heart disease interventions, we analysed trends in surgical and catheter procedures for the management of isolated shunt lesions since 2000. We found a significant rise in catheter interventions but without any discernible impact on the number of patients undergoing surgical repair, in the context of only low-level growth in the UK population. The evolution of management strategies varied according to lesion type and patient age; compare the growth of catheter interventions for ASD in adults with the minimal impact on the management of isolated VSD in infants and young children. Overall survival was excellent (>99%) at 30 days for all lesions other than VSD device closure (95.7%) and PDA ligation (95.6%), but all patient groups had ongoing attrition between 30 days and 1 year.

Few other studies have compared multicentre experiences of catheter and surgical procedures in the management of non-complex lesions in children and adults. In a Danish national study, Larsen *et al* found similar practice for the period 2003–2015, with catheter procedures commonly used for ASD (48.2%) or PDA (72.2%) but rarely in VSD (2.4%)^5^; they reported 100% 30-day and 98%–100% 1-year survival following intervention in these conditions. The IMproving Pediatric and Adult Congenital Treatment (IMPACT) registry collects data on catheter interventions from 81 US centres and reported that in 2011–2013, 73.0% of ASD device closures were performed in children (1–18 years) and 25.5% in adults^6^; this differs from our finding of 36.2% in children and 63.5% in adults for the same period and likely reflects a selection bias towards paediatric centres in their registry. In contrast, their report of 26.1% of PDA occlusions in infants and 70.0% in children is similar to our findings of 20.9% and 72.7%, respectively. The IMPACT registry does not collect data on surgical interventions and therefore cannot provide relative trends in resource utilisation over time. Conversely, Brown *et al* used NCHDA data to assess trends in 30-day mortality for all paediatric cardiac surgery in the UK and found that mortality in the lowest risk cases fell from 1.4% (95% CI 0.6 to 2.1) in 2000–2001 to 0.5% (95% CI 0.2 to 0.7) in 2009–2010 (p<0.01)^7^; outcomes of catheter interventions were not assessed.

The increase in catheter interventions over recent years has been driven by advances in non-invasive diagnostic imaging, catheter design and device technology. These have led to widened indications for catheter intervention and the expansion of paediatric and adult congenital interventional cardiology expertise.^8–10^ In contrast, there has been minimal change in operative technique or the availability of surgical services. The impact of these contrasting patterns on clinical practice has varied by the type of congenital heart defect.

### Atrial septal defect

ASD device closure has doubled over the last 16 years, with a near fourfold increase in adults (figure 2) and is now the standard of care for anatomically suitable lesions^8 10^; most of this rise reflects a genuine change in practice at established adult congenital heart centres rather than reporting of catheter procedures at additional sites, as shown in the online supplementary eFigure B. Despite this, the number of surgical ASD closures in adults has also increased, perhaps due to concerns over late device erosion in high-risk groups.^11^ In patients with a haemodynamically significant shunt but without pulmonary hypertension, closure is associated with better outcomes, fewer atrial arrhythmias, improved functional capacity and long-term survival.^12 13^ This is considered a class I (symptomatic) or class IIa (asymptomatic) indication in the recent adult American Heart Association (AHA)/American College of Cardiology guidelines,^9^ and a class I indication regardless of symptoms in the current paediatric AHA and adult European Society of Cardiology guidelines.^8 10^ The best outcome is achieved in patients undergoing closure before 25 years of age,^14^ while intervention after 40 years does not affect the frequency of atrial arrhythmias.^15^

Secundum ASDs account for 80% of cases and most are amenable to percutaneous device closure^8 10^; surgical repair is indicated primarily when the rim of septal tissue is <5 mm and therefore inadequate for device placement.^9 10^ On the other hand, sinus venosus defects (5%–10%) are usually only amenable to surgical closure due to the associated partial anomalous pulmonary venous drainage of the right lung. The apparent rise in sinus venosus closure is likely to reflect more accurate diagnosis of right heart volume loading on echocardiography and classification in reported data rather than a true increase in the incidence or rate of surgical intervention (see online supplementary eFigure D).

### Patent foramen ovale

The rapid rise in off-label PFO device closures between 2003 and 2010 was driven by the belief that it may have widespread therapeutic benefit in conditions such as migraine headache and cryptogenic stroke.^16^ However, three randomised controlled trials in recurrent migraine, including the controversial Migraine Intervention with STARFlex Technology (MIST) trial in the UK published in 2008,^17^ showed no benefit for migraine prevention^18^; routine PFO closure was not recommended by the UK National Institute for Health and Care Excellence guideline in 2010,^19^ coinciding with a sharp decline in procedures (figure 3). Similarly, in recurrent stroke, several trials failed to demonstrate clinical benefit or cost-effectiveness,^20^ leading to the withdrawal of NHS repayment in 2016. Current AHA/American Stroke Association guidelines do not support PFO closure in patients with cryptogenic stroke without evidence of a deep vein thrombosis (class III),^21^ although recent trial evidence suggests the superiority of device closure over medical therapy.^22^ It therefore is likely that device closure of PFO for the prevention of recurrent cryptogenic stroke will be performed more frequently.

### Ventricular septal defect

Surgical closure remains the standard of care for patients with an isolated haemodynamically significant VSD.^8–10^ Most perimembranous defects require closure in early childhood which may be complicated by complete atrioventricular block, requiring permanent pacemaker insertion early after surgery in 1.1% of cases.^23^ Despite advances in catheter technology, clinical concerns remain regarding device migration and delayed heart block, which may occur late after implantation in up to 5% of perimembranous VSDs and therefore present a risk of sudden death in the community.^24^ A recent meta-analysis of transcatheter device closure for perimembranous VSD in 54 studies found a pooled estimates of heart block at 1.1%, residual shunt at 15.9%, arrhythmias at 10.3% and valvular defects at 4.1%^25^; yet it was limited by the inclusion of 53 cohort studies but only one early phase clinical trial. The rate of complications is highest in smaller children who comprise most patients undergoing closure, which may explain the lack of uptake (figure 4).^26^ Device closure is considered as an alternative to surgery in paediatric and adult patients with a muscular VSD with an adequate rim of septal myocardium,^8 9^ as well as in those with a residual shunt after surgical repair. The higher 30-day mortality observed for device closure (4.1%) compared with surgery (0.6%) may reflect selection of high-risk cases considered less attractive for surgical closure or cumulative learning curves across centres.

### Patent arterial duct

The choice of technique for preventing flow in a persistent PDA is primarily determined by patient age/size. Transcatheter occlusion is performed mostly during childhood, although its use in infants increased during the study without a corresponding fall in surgery, suggesting a more proactive approach. In children, occlusion is indicated for a moderate or large PDA with a haemodynamically significant left-to-right shunt (class I), and reasonable for a small left-to-right shunt that is audible on auscultation (class IIa).^8^

Surgical ligation via a lateral thoracotomy remains the procedure of choice in neonates, who are usually premature with very low birth weight, failure to thrive and remain ventilator-dependent. Thirty-day (4.4%) and 1-year (11.0%) mortality remain high, reflecting their poor physiological state and comorbidities. However, we found the number of neonates and infants undergoing surgical ligation fell by 58.2% and 33.0%, respectively over the last 5 years (figure 5), suggesting a move away from referral for intervention and appears consistent across centres (see online supplementary eFigure E). Bixler *et al* reported a similar trend in US neonatal intensive care units with a 53% decrease in PDA ligation over the last decade, with a fall across 83% of units.^27^ This move towards conservative management reflects the growing belief that PDA closure does not improve neonatal outcomes.^28^

Transcatheter PDA occlusion is rarely performed in preterm neonates as the manufacturer currently recommends the duct occluder in patients >6 kg. A recent UK study demonstrated its feasibility and efficacy in infants <6 kg (mean 4.9±1.0 kg), achieving complete occlusion in 356/374 (95.2%) at latest follow-up (2.6±2.4 years) with 10% incidence of moderate-to-major adverse events and no procedure-related deaths.^29^ Moreover, echocardiographic-guided duct occlusion in extremely premature infants (mean 1.25 kg) has been shown to be achievable with minimal procedural morbidity.^30^ No published data directly compares outcomes of surgical ligation and transcatheter occlusion in preterm infants.^28^

### Limitations

All congenital heart units in the UK and Ireland routinely submit data to NCHDA but other cardiac centres (NHS and private) either joined during the study period or do not participate (see online supplementary eFigure A). This may underestimate the number of cases, particularly adult ASD and PFO closures performed at non-adult congenital heart disease (ACHD) centres; data on patients over 16 years therefore are likely incomplete. Reorganisation of services over the last two decades may also have affected capture as centres opened, closed or merged and may have contributed to overall trends.

As data are categorised by procedural code rather than diagnosis, heterogeneity within categories cannot be determined and patients with more complex treatment pathways may have been included, for example, catheter closure of a residual VSD postsurgery in a more complex condition, retrieval of an embolised device with surgical closure, and combined or staged procedures for multiple VSDs using combined approaches. Similarly, the use of broad age categories does not allow accurate determination of age at procedure and therefore whether there are trends towards earlier or later interventions. The focus on isolated shunt lesions excluded other conditions in which the rise in catheter interventions has impacted on surgery, such as balloon dilatation, stenting and transcatheter valve implantation.

Finally, data on survival are near-complete at 30 days but there is a significant proportion of missing data at 1 year (table 1). As NCHDA routinely perform mortality tracking via ONS for deaths registered in the UK, missing cases are likely but not proven to be alive. Excluding missing cases would lead to a significant underestimation of survival and therefore the sums of ‘known alive’ and ‘unknown’ are included as ‘assumed alive’.

## Conclusions

Trends in catheter and surgical techniques for the treatment of isolated congenital shunt lesions plot the evolution of the specialty over the last 16 years. They reflect changes in clinical guidelines, technology, expertise and reimbursement, and have distinct patterns according to lesion type and patient age. Catheter device closure has become the procedure of choice for ASD, especially in adults but the marked increase in procedures has not reduced surgical workload. On the other hand, it has had minimal impact on the management of VSD during childhood; this may change with improving device technology and techniques, and accumulating evidence of better safety and efficacy. PFO closure underwent a period of rapid growth with widespread clinical adoption before evidence from several clinical trials failed to show benefit and it was not recommended in national guidelines, leading to a sharp decline. Finally, there has been little change in the management of PDA, although the coming years will determine the extent of the rise of catheter occlusion in small children and decline of surgical ligation in premature infants. 

**Key messages**

**What is already known on this subject?**

- Surgical repair has been the mainstay of treatment for congenital heart disease in children and adults.
- With advances in technology and availability, transcatheter procedures are commonly used in many centres for isolated shunt defects with reduced early morbidity.

**What might this study add?**

- In this study of 40 911 children and adults undergoing intervention from 2000 to 2016, the use of catheter and surgical techniques varied according to lesion type and patient age.
- Catheter intervention has become the procedure of choice for atrial septal defect and patent foramen ovale, but there has been little change in the management of ventricular septal defect or patent arterial duct.
- Overall survival was excellent (>99%) at 30 days for all lesions other than ventricular septal defect device closure (95.7%) and patent arterial duct ligation (95.6%), but all patient groups had ongoing attrition between 30 days and 1 year.

**How might this impact on clinical practice?**

- Transcatheter interventions for isolated shunt lesions have increased over recent years but with little impact on surgical workload.
- With advances in technology and expansion of indications, it is likely that catheter interventions will continue to increase but surgery remains an important treatment modality for isolated defects.
