# Supplementary material for: Trends in surgical and catheter interventions for isolated congenital shunt lesions in the UK and Ireland
Source: Heart. 2019 Feb 16;105(14):1103–8. doi: 10.1136/heartjnl-2018-314428 (PMC6613741; doi:10.1136/heartjnl-2018-314428)
Supplement: Supplementary file 1 [file heartjnl-2018-314428supp001.pdf]

SUPPLEMENT

Trends in surgical and catheter interventions for isolated congenital shunt lesions in UK & Ireland

eTable 1. Atrial septal defect catheter device closure by age group and year.

Year	Neonates	Infants	Children	Adults	Total
2000-01	1	1	211	110	323
2001-02	2	1	118	169	290
2002-03	2	0	202	210	414
2003-04	3	1	272	258	534
2004-05	0	4	218	292	514
2005-06	2	0	244	362	608
2006-07	1	2	229	415	647
2007-08	1	2	187	364	554
2008-09	1	6	217	432	656
2009-10	0	3	208	410	621
2010-11	0	2	229	413	644
2011-12	0	0	242	432	674
2012-13	0	5	245	423	673
2013-14	0	6	259	466	731
2014-15	0	4	245	423	672
2015-16	0	3	286	419	708
**Total**	**13**	**40**	**3,612**	**5,598**	**9,263**

eTable 2. Atrial septal defect surgical closure, including sinus venosus defect, by age group and year.

Year	Neonates	Infants	Children	Adults	Total
2000-01	4	16	214	65	299
2001-02	2	11	168	69	250
2002-03	2	16	189	80	287
2003-04	1	24	225	79	329
2004-05	3	36	192	91	322
2005-06	0	17	189	166	372
2006-07	1	15	229	109	354
2007-08	2	13	216	116	347
2008-09	2	17	229	145	393
2009-10	2	9	214	166	391
2010-11	0	19	231	180	430
2011-12	4	18	225	130	377
2012-13	2	26	243	149	420
2013-14	1	10	225	176	412
2014-15	1	13	240	141	395
2015-16	0	16	198	163	377
**Total**	**27**	**276**	**3,427**	**2,025**	**5,755**

eTable 3. Patent foramen ovale catheter device closure by age group and year.

Year	Neonates	Infants	Children	Adults	Total
2000-01	0	0	0	1	1
2001-02	0	0	0	6	6
2002-03	0	0	0	11	11
2003-04	1	0	0	52	53
2004-05	0	0	6	115	121
2005-06	1	1	4	276	282
2006-07	1	0	8	413	422
2007-08	0	0	7	558	565
2008-09	0	1	6	646	653
2009-10	0	0	3	899	902
2010-11	0	0	5	797	802
2011-12	1	0	4	734	739
2012-13	0	0	8	525	533
2013-14	0	0	7	510	517
2014-15	0	0	5	375	380
2015-16	0	0	5	473	478
**Total**	**4**	**2**	**68**	**6,392**	**6,465**

eTable 4. Ventricular septal defect catheter device closure by age group and year.

Year	Neonates	Infants	Children	Adults	Total
2000-01	1	2	6	2	11
2001-02	0	0	3	3	6
2002-03	0	1	16	8	25
2003-04	0	6	36	15	57
2004-05	0	1	42	16	59
2005-06	0	4	41	24	69
2006-07	1	1	34	19	55
2007-08	0	3	23	21	47
2008-09	0	4	21	15	40
2009-10	0	1	20	17	38
2010-11	0	7	18	17	42
2011-12	0	1	9	10	20
2012-13	0	0	15	6	21
2013-14	1	2	20	10	33
2014-15	0	7	18	11	36
2015-16	0	5	27	13	45
**Total**	**3**	**45**	**349**	**208**	**604**

eTable 5. Ventricular septal defect surgical closure, including multiple VSDs, by age group and year.

Year	Neonates	Infants	Children	Adults	Total
2000-01	7	262	81	7	357
2001-02	11	204	73	8	296
2002-03	14	256	94	7	371
2003-04	14	237	96	4	351
2004-05	15	226	70	9	320
2005-06	7	259	67	11	344
2006-07	2	239	83	4	328
2007-08	5	241	85	6	337
2008-09	6	229	65	15	315
2009-10	2	279	94	10	385
2010-11	5	282	67	5	359
2011-12	8	329	79	5	421
2012-13	5	283	76	8	372
2013-14	3	263	86	10	362
2014-15	2	267	78	11	358
2015-16	1	271	66	9	347
**Total**	**107**	**4,127**	**1,260**	**129**	**5,623**

eTable 6. Patent arterial duct catheter device closure by age group and year.

Year	Neonates	Infants	Children	Adults	Total
2000-01	2	28	319	14	363
2001-02	0	20	291	48	359
2002-03	5	40	287	16	348
2003-04	2	56	336	38	432
2004-05	0	84	350	27	461
2005-06	3	84	386	56	529
2006-07	0	90	380	31	501
2007-08	1	104	384	33	522
2008-09	2	101	378	30	511
2009-10	0	88	351	23	462
2010-11	1	111	428	28	568
2011-12	1	134	423	44	602
2012-13	1	159	413	24	597
2013-14	1	120	461	41	623
2014-15	1	129	428	34	592
2015-16	1	107	382	15	505
**Total**	**21**	**1,455**	**5,997**	**502**	**7,975**

eTable 7. Patent arterial duct surgical ligation by age group and year.

Year	Neonates	Infants	Children	Adults	Total
2000-01	90	121	13	0	224
2001-02	107	132	13	0	252
2002-03	111	174	11	0	296
2003-04	109	179	5	0	293
2004-05	102	199	3	0	304
2005-06	114	224	8	0	346
2006-07	117	221	13	1	352
2007-08	113	161	18	0	292
2008-09	137	224	5	0	366
2009-10	132	242	10	0	384
2010-11	177	273	6	0	456
2011-12	147	240	9	0	396
2012-13	137	230	5	0	372
2013-14	130	206	15	0	351
2014-15	94	180	5	0	279
2015-16	74	183	6	0	263
**Total**	**1,891**	**3,189**	**145**	**1**	**5,226**

eTable 8. 30-day survival by year quartile and procedure.

Defect	Intervention	Cases	Overall survival	2000-2004		2004-2008		2008-2012		2012-2016	
				Alive	Dead	Alive	Dead	Alive	Dead	Alive	Dead
ASD, n (%)	Catheter	9,263	9,245 (99.8)	1,560 (99.9)	1 (0.1)	2,317 (99.7)	6 (0.3)	2,589 (99.8)	5 (0.2)	2,779 (99.8)	2 (0.2)
	Surgery	5,755	5,727 (99.5)	1,158 (99.4)	7 (0.6)	1,386 (99.4)	8 (0.6)	1,585 (99.6)	6 (0.4)	1,598 (99.6)	5 (0.3)
PFO, n (%)	Catheter	6,465	6,451 (99.8)	71 (100)	0	1,387 (99.8)	3 (0.2)	3,090 (99.8)	6 (0.2)	1,903 (99.7)	5 (0.3)
VSD, n (%)	Catheter	604	578 (95.7)	92 (92.9)	7 (7.1)	221 (96.1)	8 (3.5)	140 (96.4)	5 (3.6)	130 (96.3)	5 (3.7)
	Surgery	5,623	5,588 (99.4)	1,357 (98.7)	18 (1.3)	1,322 (99.5)	6 (0.5)	1,474 (99.6)	5 (0.3)	1,435 (99.7)	4 (0.3)
PDA, n (%)	Catheter	7,975	7,962 (99.8)	1,498 (99.7)	4 (0.3)	2,011 (99.9)	2 (0.1)	2,141 (99.9)	2 (0.1)	2,312 (99.8)	5 (0.2)
	Surgery	5,226	4,997 (95.6)	995 (93.4)	69 (6.5)	1,244 (96.1)	50 (3.9)	1,545 (96.4)	57 (3.6)	1,213 (95.9)	52 (4.1)
Overall, n (%)	Both	40,911	40,548 (99.1)	6,731 (98.4)	106 (1.6)	9,888 (99.1)	83 (0.8)	12,559 (99.3)	86 (0.7)	11,370 (99.3)	78 (0.7)

ASD, atrial septal defect; PDA, patent arterial duct; PFO, patent foramen ovale; VSD, ventricular septal defect.

eFigure A. Trends in the number of centres reporting data to NCHDA by type of intervention and age group.

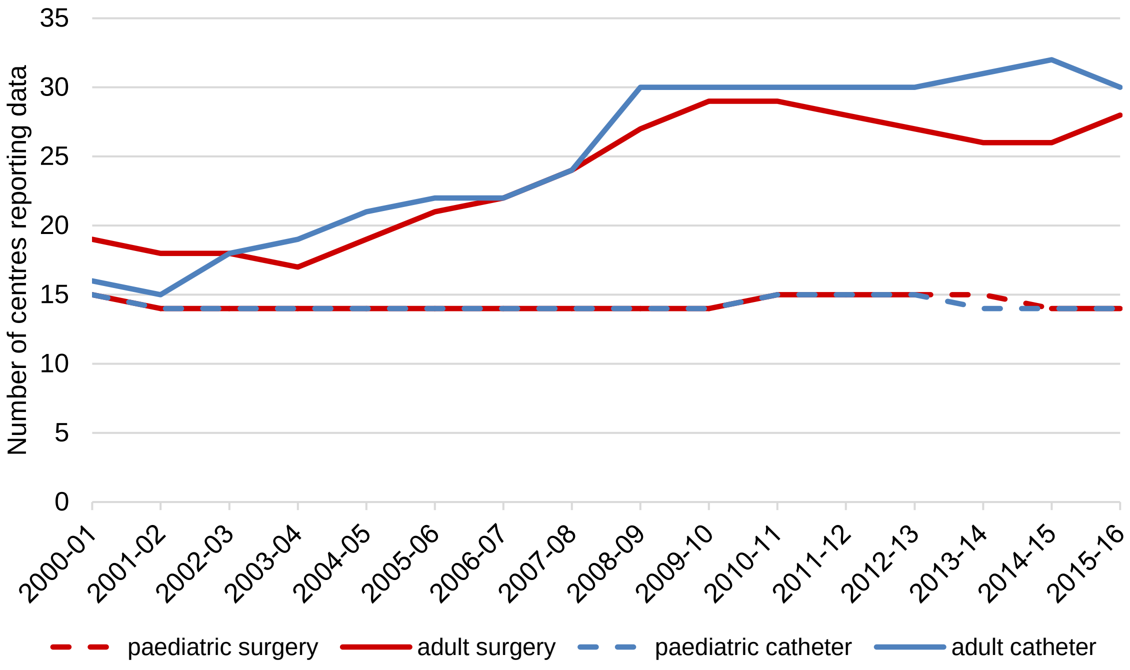

NCHDA, National Congenital Heart Disease Audit.

eFigure B. Trends in the number of catheter atrial septal defect closures in adults by established ACHD centres (reporting case volume since 2000) and additional centres.

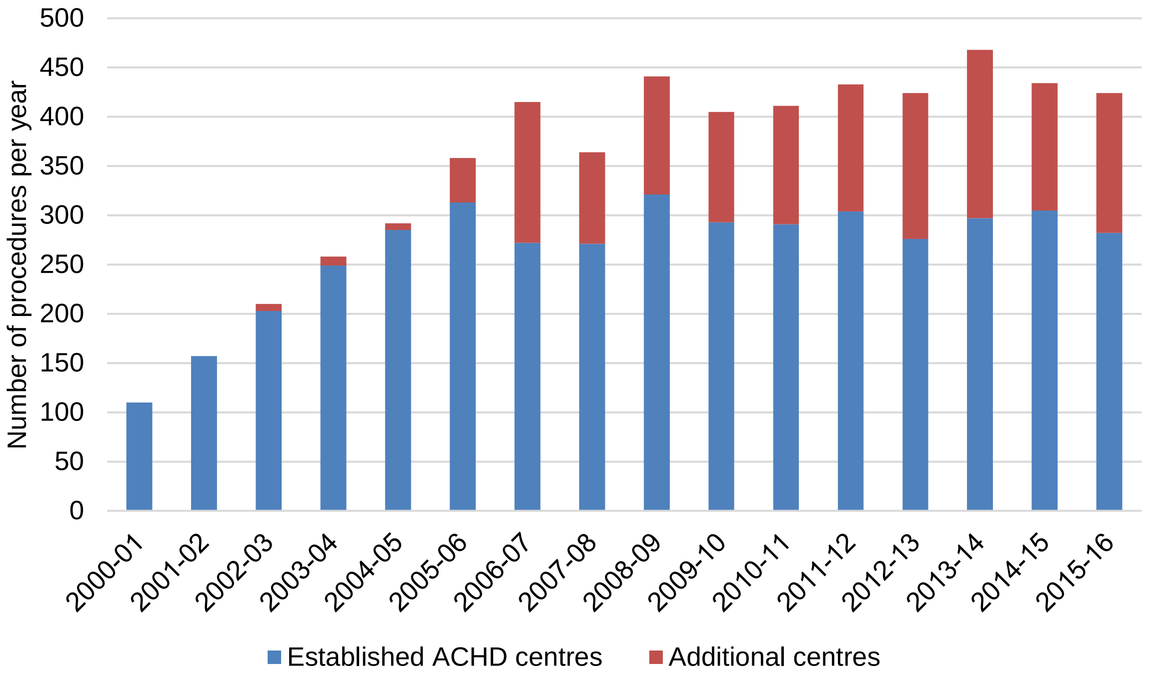

ACHD, congenital heart disease.

eFigure C. Trend in the ratio of catheter to surgical procedures for atrial septal defect.

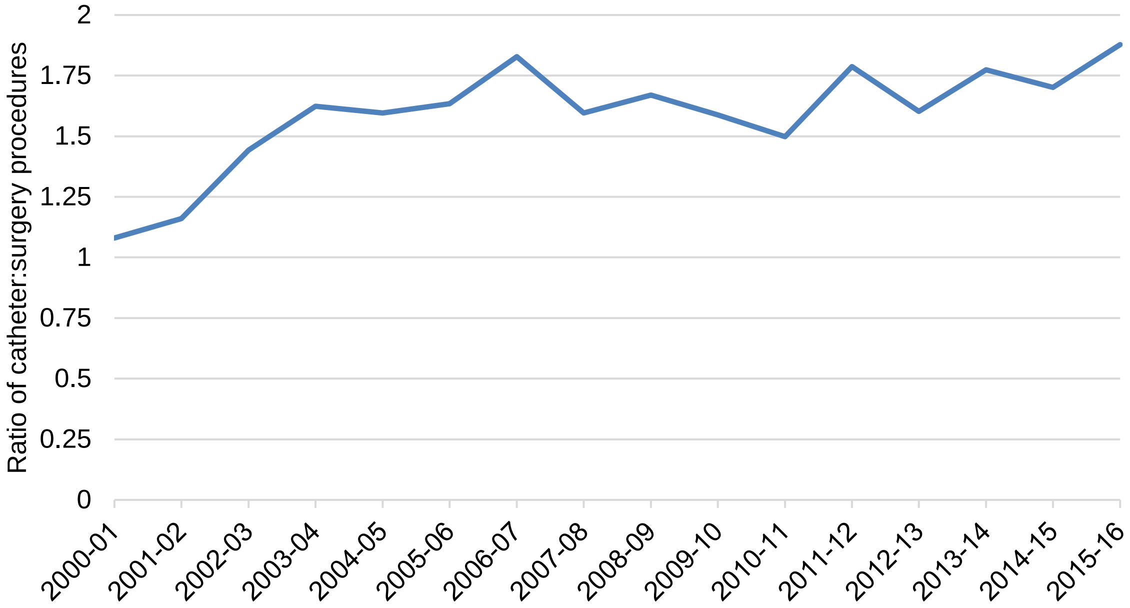

eFigure D. Trends in the number of surgical atrial septal defect closures over time, recorded as ‘ASD repair’ or ‘Sinus venosus ASD and-or PAPVC repair’.

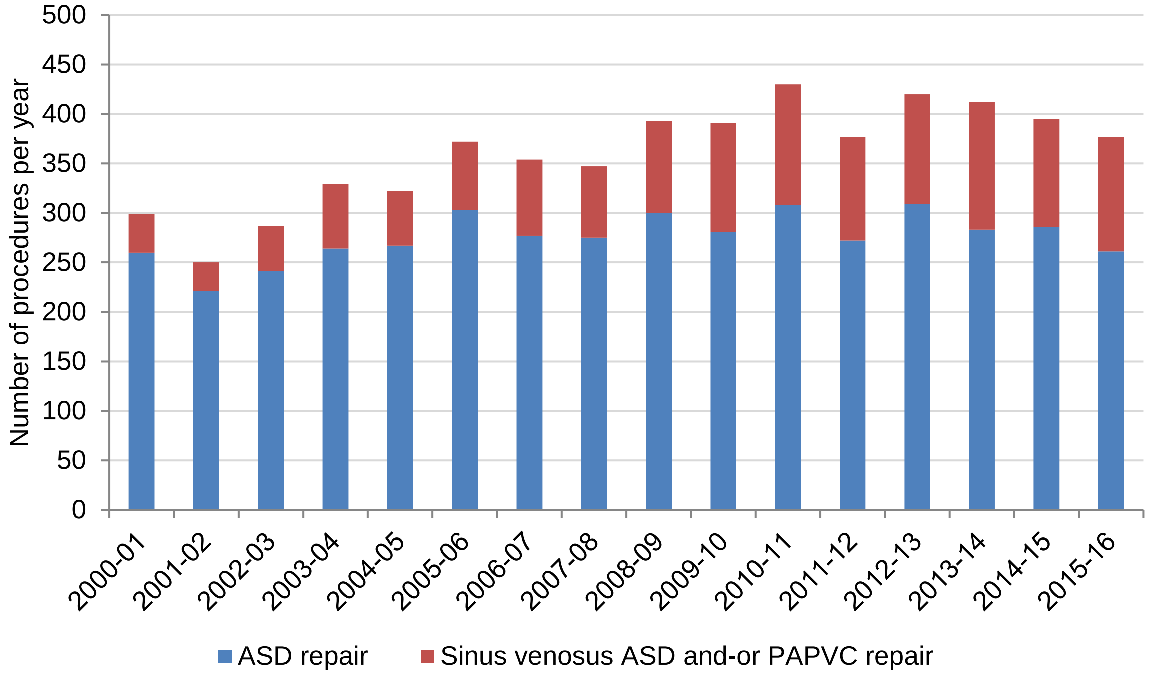

ASD, atrial septal defect; PAPVC, partial anomalous pulmonary venous connection.

eFigure E. Number of surgical ligations of a patent arterial duct since 2010 by centre.

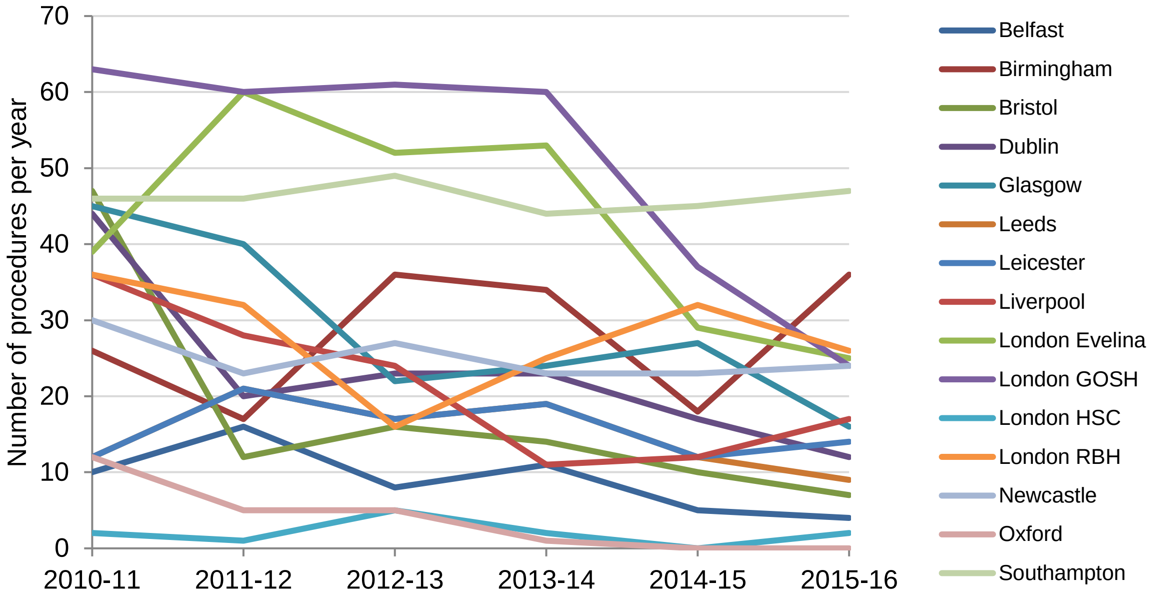

GOSH, Great Ormond Street Hospital; HSC, Harley Street Clinic; RBH, Royal Brompton Hospital.
